# Life Course Vaccination Continuum (LCVAC): An Expert Consensus on Life Course Vaccination in India

**DOI:** 10.7759/cureus.110007

**Published:** 2026-05-31

**Authors:** Parvaiz A Koul, Agam Vora, Mangesh Tiwaskar, Rahul Bhargava, Raja Dhar, V. R. Ramasubramanian, Vijay Kher, Vivek Nangia, Vivekanand Jha, Trayambak Dutta, Manish Mahajan

**Affiliations:** 1 Internal Medicine and Pulmonary Medicine, Sher-i-Kashmir Institute of Medical Sciences, Srinagar, IND; 2 Pulmonology, Vora Clinic, Mumbai, IND; 3 Diabetes and Endocrinology, Shilpa Medical Research Centre, Mumbai, IND; 4 Haematology, Fortis Memorial Research Institute and Hospital, Gurugram, IND; 5 Pulmonology, The Calcutta Medical Research Institute, Kolkata, IND; 6 Infectious Disease, Apollo Hospitals, Chennai, IND; 7 Nephrology, Epitome Kidney Urology Institute and Lions Hospital, New Delhi, IND; 8 Pulmonary and Critical Care Medicine, Max Hospital, New Delhi, IND; 9 Nephrology, George Institute for Global Health, New Delhi, IND; 10 Infectious Disease, Zydus Lifesciences, Ahmedabad, IND; 11 Medical Affairs, Zydus Lifesciences, Ahmedabad, IND

**Keywords:** immunisation policy, indian vaccination programs, life course vaccination, preventive health strategies, public health, vaccine accessibility

## Abstract

India's immunization program focuses predominantly on children, leaving adults vulnerable to vaccine-preventable diseases (VPDs). The COVID-19 pandemic further disrupted routine vaccination. A life course approach (LCA) is needed to address these gaps. However, implementation of adult vaccination programs in low- and middle-income countries (LMICs) remains challenged by inequities in healthcare access, infrastructural limitations, and variable awareness regarding adult immunization.

A 16-member expert panel from pulmonology, geriatrics, oncology, obstetrics, nephrology, diabetology, and life sciences reviewed literature (2014-2023) from MEDLINE, Scopus, and Cochrane databases. Using a structured, evidence-based grading approach, the panel formulated consensus recommendations for adult vaccination in India.

The Life Course Vaccination Continuum (LCVAC) proposes three target groups: adults aged 18-59 years with comorbidities; elderly ≥60 years with or without comorbidities; and healthy adults aged 18-60 years. Key recommended vaccines include Tdap (every 10 years), influenza (annual), pneumococcal (PCV13/PPSV23 series), zoster (for ≥50 years), hepatitis B, meningococcal, and hepatitis A (risk-based). Major barriers identified include vaccine hesitancy, cost, access limitations, and inadequate provider prioritization.

Life Course Vaccination Continuum provides the first comprehensive expert consensus on life course vaccination in India. The framework is intended to complement broader public health strengthening efforts, and its successful implementation will require vaccine awareness campaigns, healthcare professional education, equitable access strategies, and the establishment of dedicated adult vaccination centers nationwide.

## Introduction and background

Vaccination remains the most effective intervention in preventing and controlling the spread of infectious diseases and reducing morbidity and mortality [1,2]. The developmental pathway of vaccines involves exhaustive scrutiny and approval processes to ensure adherence to standards of safety, efficacy, and quality [3].

Projections indicate that by the year 2050, the global population of adults aged over 65 will be twice that of the under-five population, with a significant concentration in low- and middle-income countries [4]. The adult population, encompassing individuals aged more than 18 years, and adolescents, represents a critical target group for ongoing immunisation programmes. Acknowledging this, the World Health Organization's Strategic Advisory Group of Experts (SAGE) for the Global Programme for Vaccines and Immunization (GPV) underscores the imperative to extend immunisation activities beyond the paediatric age group, whether integrated into routine services or as part of disease elimination and eradication strategies [5].

As of 2024, vaccination coverage in India has shown significant progress, yet challenges remain in achieving universal immunisation. The country has made substantial strides in its vaccination programmes, particularly through initiatives like the Universal Immunisation Programme (UIP), which aims to provide comprehensive immunisation to infants and children [6]. Recent statistics indicate that approximately 94% of children receive the recommended vaccines by their first year, reflecting significant achievements of the introduced programs and implementation. Nevertheless, disparities persist in rural and underserved areas [7]. The COVID-19 pandemic has further exacerbated the challenges in vaccination efforts, leading to temporary declines in coverage for routine immunisations. Although reports suggest that India supplied over 2.4 billion doses of COVID-19 vaccines globally, ensuring consistent access to vaccines for other preventable diseases remains a priority [8].

Although life-course vaccination strategies have been successfully implemented in several high-income countries, translating similar models into low- and middle-income countries (LMIC) settings presents unique challenges. Inequities in universal health coverage, inadequate adult vaccination infrastructure, fragmented health systems, limited awareness regarding adult immunization, financial constraints, and competing healthcare priorities may significantly affect the sustainability and reach of vaccination programs in these regions. Consequently, vaccination alone cannot address broader systemic healthcare gaps unless supported by robust healthcare delivery systems, surveillance mechanisms, policy-level prioritization, healthcare workforce training, and equitable access frameworks.

In this context, the Life Course Vaccination Continuum (LCVAC) framework is intended not as a standalone intervention, but as a preventive healthcare approach that can complement ongoing efforts toward strengthening public health systems and healthy ageing strategies in India. The framework aims to provide evidence-based expert guidance to support risk-based and age-based immunization practices while acknowledging the practical realities and implementation challenges within resource-constrained settings.

Although various global guidelines/recommendations are in place for vaccination, specific Indian recommendations for clinicians practicing in the country are lacking. Recently, the Association of Physicians of India (API) has established guidelines for vaccination in India, particularly focusing on adults, to address the lack of consensus on immunization strategies. The API guidelines for vaccination in India have several limitations exemplified by a lack of uniform agreement among national organizations regarding adult vaccination strategies, leading to inconsistent implementation and awareness. Insufficient surveillance data on vaccine-preventable diseases (VPDs) hampers effective planning. Vaccine hesitancy, influenced by cultural factors, limits uptake, while logistical challenges hinder accessibility, especially in rural areas. Additionally, inadequate advocacy and public education about the benefits of adult vaccinations contribute to low utilization of available services. Addressing these gaps is crucial for improving adult vaccination rates in India.

To address the limitations of existing adult vaccination guidelines in India, the Life Course Vaccination Continuum (LCVAC) was established. This multidisciplinary advisory committee comprises 16 experts from pulmonology, geriatrics, oncology, obstetrics, nephrology, diabetology, and the life sciences industry. The LCVAC initiative focuses on three age groups: adults aged 18-59 years with comorbidities; elderly adults ≥60 years with or without comorbidities; and healthy adults aged 18-60 years. Its core mission is organized around three pillars: vaccine awareness, vaccine education for healthcare professionals, and the establishment of dedicated adult vaccination centers across India. The objectives of the LCVAC policy are: (1) To establish a comprehensive vaccination program, (2) To conduct educational campaigns and workshops to address hesitancy and promote immunization, (3) To offer clinical skills training to healthcare professionals, and (4) To collaborate with community leaders and advocacy groups to address specific needs, while also establishing a robust policy development process involving experts, evidence-based deliberation, and stakeholder consultations.

## Review

Methodology

In India, drafting an effective life course vaccination (LCV) policy necessitates a well-defined yet adaptable policy development process guided by a dedicated Life Course Vaccination Continuum.

Expert Panel Composition and Selection

The committee, composed of diverse experts - epidemiologists, immunologists, paediatricians, public health specialists, and representatives from advocacy groups - serves as the cornerstone of policy formulation. There were 16 expert members and four moderators. These 16 experts are key opinion leaders (KOLs) and were selected based on their respective areas of expertise and convened to achieve consensus. The expert panel members included experts from the disciplines of Pulmonology, Geriatrics, Oncology, Obstetrics, Nephrology, Diabetology, and the Life Sciences industry from various parts of India. The therapeutic areas were discussed thoroughly by the expert panel, resulting in the achievement of a consensus through agreement. Moderators facilitated discussions but were not involved in the consensus determination.

Literature Search Strategy

Although this expert consensus was not designed as a formal systematic review, the literature review process was guided by key principles of an adapted Preferred Reporting Items for Systematic Reviews and Meta-Analyses (PRISMA)-informed approach, including predefined databases, search period, search terms, language restrictions, prioritization of Indian evidence where available, and transparent documentation of the evidence selection and consensus process.

These experts critically analysed existing literature, including randomized clinical trials, systematic reviews, and meta‑analyses, as well as key global and Indian guidelines and recommendations on adult vaccination, emphasizing the disease burden of VPDs, vaccine availability, immunization coverage, and modifiable challenges across various age groups. Evidence to frame specific questions was obtained through a literature search of MEDLINE (via PubMed), Scopus, and Cochrane‑indexed databases on adult vaccination published between January 2014 and December 2023.

The keywords for the literature search were as follows: definitions, epidemiology, India, pneumonia, influenza vaccines, adult vaccination, influenza vaccine, new vaccines, optimizing schedules, COVID-19, Measles, mumps, rubella, Human papillomavirus, RZV, VAR, Tdap, storage, prevention, mortality, impact, elderly, serotype, safety, immunization coverage, disease burden patterns, vaccine availability, immunogenicity, comorbid conditions, immunosenescence, mass gatherings, guidelines, and recommendations. Based on these keywords, a series of clinical questions on adult vaccination was formulated. These questions were formulated following discussions about several factors unique to the Indian context. The search was restricted to English-language publications. Studies from any geographic region were included, but Indian-specific data were prioritized when available.

Institutional review board approval was not required for this expert consensus document, as no primary data were collected from human subjects.

Consensus Process

Literature reviews and discussions for each disease were coordinated by group chairs and were well documented by rapporteurs. Discussions regarding the grading of evidence and recommendations were held independently in four parallel‑group sessions where various therapy areas were discussed, grounded in a review of the relevant literature. These discussions were subsequently followed by a joint plenary session involving all experts, during which a collective consensus was reached. Consensus was predefined as agreement by ≥80% of participants. Notably, as all KOLs were experienced physician vaccinators actively incorporating vaccination into their routine clinical practice, unanimous agreement (100% consensus) was ultimately achieved. There is no conflict of interest to be declared.

Evidence Grading

The Grading of Recommendations Assessment, Development and Evaluation (GRADE) framework is an internationally recognized, transparent methodology for rating the quality of evidence and the strength of clinical recommendations. Originally developed by the GRADE Working Group, it classifies the certainty of evidence based on factors such as study design, risk of bias, inconsistency, indirectness, and imprecision. The strength of a recommendation is then determined by weighing evidence quality alongside the balance of benefits and harms, patient values and preferences, resource implications, and feasibility. Given the contextual needs of this Indian expert consensus, including heterogeneity of available evidence, limited India-specific data for several vaccine-preventable diseases, and practical realities of clinical decision-making in resource-variable settings, a modified GRADE framework was adopted, as described by Dhar et al. [9]. This adaptation retains the core principles of evidence hierarchization and recommendation grading while allowing for pragmatic expert judgment in areas where high-quality randomized evidence is lacking or absent.

**Figure 1:**
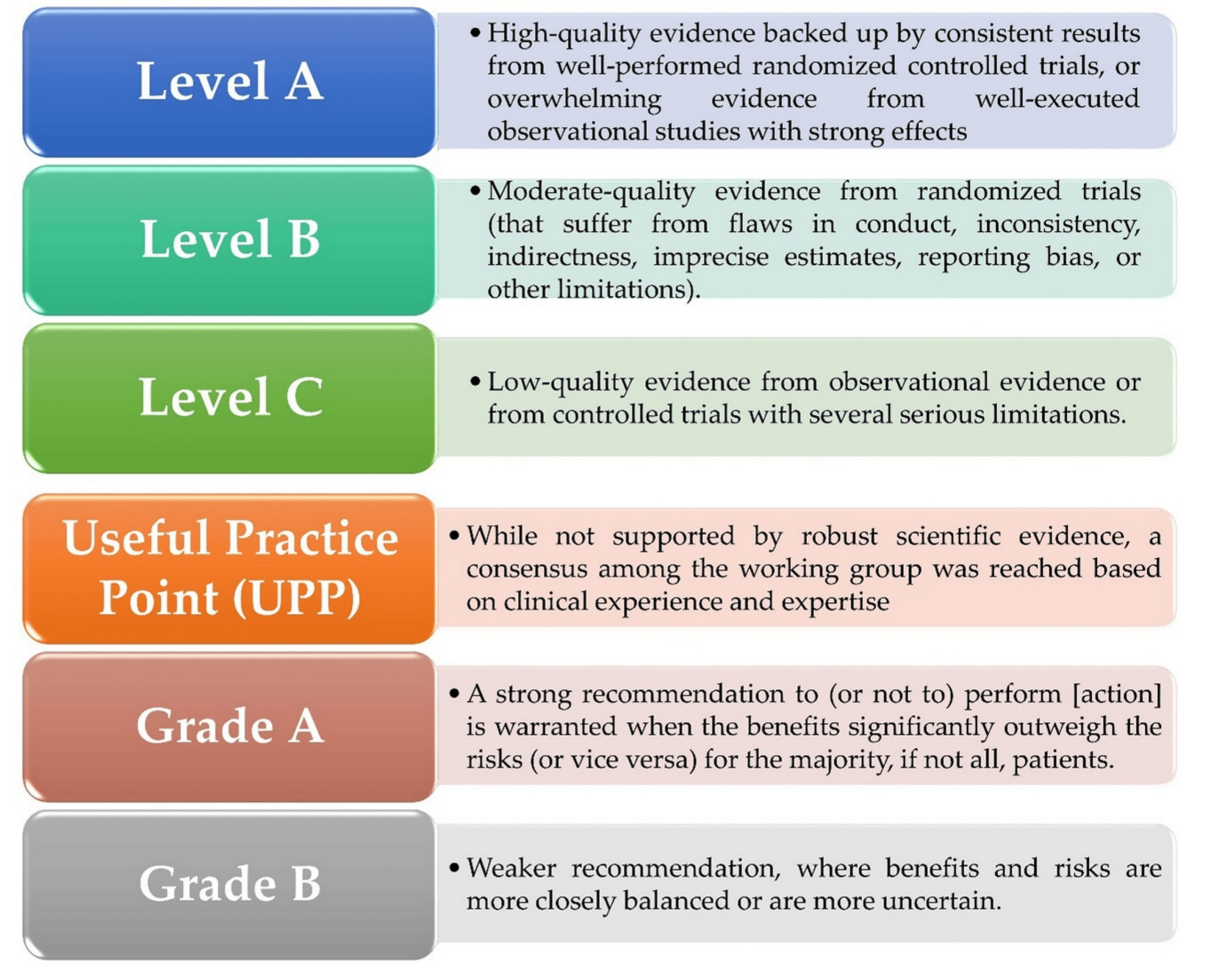
Classification of the level of evidence and grading of clinical recommendations Source: Adapted from Dhar et al. [9].

A consensus‑based approach was used to arrive at the final decision on clinical recommendations in the joint session. The modified grade system was utilized for categorizing the level of evidence as 1, 2, 3, or Useful Practice Point (UPP) (Figure 1, adapted from Dhar et al. [9]). The strength of recommendation was graded as A or B, based on the level of evidence. Grade A should be interpreted as “recommended,” while Grade B as “suggested.” Reaching consensus among the multidisciplinary panel was initially challenging due to differing clinical priorities, interpretations of evidence, and practice perspectives across specialties. However, these differences were effectively resolved through structured discussions, iterative clarification, and collaborative engagement. Facilitated moderation and mutual respect enabled the group to align on key concepts, balance risk-benefit considerations, and ultimately arrive at unified, patient-centered recommendations. All aspects related to practicality, implementation, costs, and clinical feasibility in the region at various health‑care levels were duly considered while formulating the clinical recommendations. This consensus process was not registered prospectively, but the methodology was documented in real time and reviewed by all co-authors.

Adult vaccination: current status and LCVAC recommendation

In contrast to the Pediatrics Immunization Guidelines, there is significant regional variation in adult vaccination practices within India. The most widely adopted guidelines for adult immunization in India include those from the World Health Organization, the Geriatric Society of India, the Advisory Committee on Immunization Practices (ACIP) of the United States Centre for Disease Control and Prevention (CDC), Indian Chest Society and National College of Chest Physicians (ICS/NCCP) guidelines on pneumococcal vaccination, the Association of Physicians of India's Expert panel, the Research Society for Study of Diabetes in India, the Indian Society of Nephrology, and the Indian Medical Association (IMA) [10-13].

In this section, a detailed catalogue of recommended vaccines by the LCVAC panel is presented, encompassing diseases such as COVID-19, influenza, pneumococcal infection, herpes zoster, meningococcal disease, and others.

Tetanus, Diphtheria, and Pertussis (Tdap) Vaccine

Tdap vaccination is a critical preventive measure against three serious bacterial infections: tetanus, diphtheria, and pertussis [14]. The global disease burden of tetanus decreased substantially from 1990 to 2019, with neonatal tetanus being most serious in low sociodemographic index (SDI) areas. The proportion of tetanus cases among the elderly is highest in high SDI regions [15]. Seroprevalence data suggest immunity levels are below herd immunity thresholds in several Asian countries in older children and adolescents for diphtheria and pertussis [16]. Tdap vaccines are recommended for older children, adolescents, and adults, with boosters every 10 years. Pregnant women should receive Tdap during each pregnancy to protect themselves and pass antibodies to their newborns. Tdap vaccination has significantly reduced the incidence of these diseases. Side effects are generally mild, such as fever and soreness at the injection site. Tdap is a safe and effective way to prevent these potentially life-threatening illnesses. While the primary focus has been on the DPT (diphtheria, pertussis, tetanus) vaccine for infants, the Indian Ministry of Health and Family Welfare (MoHFW) has recognized the importance of Tdap for older children and adults [17]. Limited data is available on the epidemiology of tetanus, diphtheria, and pertussis in older Indian children due to weak surveillance systems. Outbreaks and rising incidence have occurred in this age group in recent years, highlighting the need for booster vaccinations [18].

The recommendations of LCVAC are as follows.

Dosage and Administration: One dose of Tdap, followed by Tdap boosters every 10 years.

Target Population: Adults over 18 years who have not received a Tdap vaccine before, followed by Tdap boosters (Grade A, Level A).

Special Considerations: Tdap is particularly important for the elderly who may have close contact with infants (Grade A, Level A).

Pregnancy: The Tdap vaccine is safe during pregnancy. Recommended during each pregnancy, preferably in the early part of gestational weeks 27-36 (Grade A, Level A).

Influenza Vaccine

The trivalent vaccine is a crucial public health intervention that significantly reduces the incidence of influenza and its complications. It is recommended annually for everyone aged six months and older due to the virus's ability to mutate, necessitating yearly updates to the vaccine formulation [19,20]. Studies indicate that flu vaccination can decrease the risk of flu illness by 40% to 60%, prevent millions of illnesses, and reduce hospitalizations and deaths associated with influenza [21]. Over the last five years, influenza has significantly impacted public health in India, with approximately 27,825 deaths reported in children under five in 2016 [22]. In 2020, deaths attributed to influenza and pneumonia reached 392,425, accounting for 4.63% of total deaths in the country [23]. From 2021 to 2023, while specific mortality figures are less documented, ongoing surveillance highlighted notable cases of H1N1 and H3N2, with confirmed deaths from H3N2 reported [24]. This underscores the continued burden of influenza and the need for enhanced surveillance and vaccination strategies to mitigate its impact. Additionally, vaccinated individuals who contract the virus often experience milder symptoms and shorter hospital stays compared to unvaccinated individuals, underscoring the vaccine's importance in protecting vulnerable populations. The MoHFW issues guidelines for healthcare providers and the public on influenza prevention, including vaccination recommendations for high-risk groups like the elderly, children, pregnant women, and those with chronic illnesses.

The WHO’s 2025-26 recommendations of NH strainshighlight the persistent absence of naturally occurring B/Yamagata lineage viruses since March 2020. As a result, the inclusion of a B/Yamagata antigen in the quadrivalent influenza vaccine is no longer considered necessary. Therefore, the trivalent influenza vaccine has been recommended over the quadrivalent influenza vaccine by the US CDC, followed by the Indian National Centre for Disease Control (NCDC) from the current NH 2025-26 season [25,26].

The following prefilled syringes of trivalent vaccines are available in India: a) Trivalent inactivated vaccine, split virion and subunit vaccines, and b) Live, attenuated nasal spray (lyophilized).

Split-virion vaccines disrupt the influenza virus with a detergent, retaining more internal proteins, which can elicit both a cellular and antibody response, leading to potentially greater effectiveness, especially in older adults. Subunit vaccines are more purified, removing most internal components, and rely mainly on the surface proteins (HA and NA) to create an antibody response. Consequently, split-virion vaccines may be more effective, while subunit vaccines might have fewer side effects due to their higher purification. For immunocompromised adults, a split-virion inactivated influenza vaccine is preferred. For healthy adults, either split-virion or subunit trivalent vaccines are acceptable [20].

Several studies have demonstrated the safety and efficacy of administering pneumococcal and trivalent vaccines concurrently.

The recommendations of LCVAC are as follows.

Dosage and Administration: Annual vaccination is recommended for all individuals, with particular emphasis on those at a higher risk for influenza complications. The trivalent vaccine offers protection against two strains of influenza A virus and one strain of influenza B virus. The influenza vaccine is recommended for yearly administration [27].

Target Population: All adults aged 50 and older are a priority group for vaccination, as they may be more likely to have chronic medical conditions that put them at higher risk of severe influenza illness; complications, hospitalizations, and deaths are most common in adults aged 65 years and older [28]. Pregnant women, children under 5 years, and individuals with chronic medical conditions (e.g., asthma, diabetes) are also recommended. A single dose is recommended annually, especially before the flu season, which varies regionally across India (Grade A, Level A).

Co-administration of Various Vaccines: Consequently, the concomitant use of pneumococcal and influenza vaccine (trivalent) is recommended (Grade A, Level B) [28,29].

Pneumococcal Vaccine

In 2020, pneumonia and influenza deaths accounted for approximately 392,425 fatalities, representing 4.63% of total deaths in the country. The estimated national pneumonia case fatality rate was about 0.38%, with severe pneumonia at 2.26%. While the overall incidence has declined since 2000, India still accounts for around 20% of childhood pneumonia deaths globally [23,30]. Efforts to improve vaccination coverage and address risk factors like malnutrition and air pollution are critical for reducing pneumonia mortality in the future. Vaccines are crucial for preventing invasive pneumococcal disease (IPD) caused by *Streptococcus pneumoniae *bacteria. The 23-valent pneumococcal polysaccharide vaccine (PPV23) and 13-valent pneumococcal conjugate vaccine (PCV13) are recommended for high-risk individuals aged 2 years and older [31,32]. PCV13 may provide better protection due to its conjugate properties, allowing for memory B-cell response in addition to the usual T-cell-mediated response to the vaccination. Pneumococcal vaccination has significantly reduced IPD incidence, despite serotype replacement. Uptake of pneumococcal vaccines remains suboptimal, especially among high-risk groups, highlighting the need for improved awareness and access. Pneumococcal vaccination is a safe and effective way to prevent serious pneumococcal infections. The Indian Chest Society (ICS) guidelines recommend administering the PPSV23 vaccine to all adults over 65 years and suggest a sequence of PCV13 followed by PPSV23 for individuals with immunocompromising conditions. The Indian Association of Occupational Health Guidelines for Working Adults (IAOH) emphasizes the importance of pneumococcal vaccination for high-risk groups and supports the use of both PCV13 and PPSV23, particularly for older adults and those with chronic health conditions [13]. The MoHFW has been actively promoting the importance of pneumococcal vaccination through public awareness campaigns [33,34].

The recommendations of LCVAC are as follows.

Vaccine Type: Pneumococcal polysaccharide vaccine (PPSV23) provides protection against 23 serotypes of *Streptococcus pneumoniae*, viz., 1, 2, 3, 4, 5, 6B, 7F, 8, 9N, 9V, 10A, 11A, 12F, 14, 15B, 17F, 18C, 19A, 19F, 20, 22F, 23F, and 33F.

Pneumococcal conjugate vaccine 13 (PCV13) provides protection against 13 serotypes of *S. pneumoniae,* viz., 1, 3, 4, 5, 6A, 6B, 7F, 9V, 14, 18C, 19A, 19F, and 23F. Pneumococcal conjugate vaccine 15 (PCV15) provides protection against 15 serotypes of *S. pneumoniae,* viz., 1, 3, 4, 5, 6A, 6B, 7F, 9V, 14, 18C, 19A, 19F, 22F, 23F, and 33F. 

Pneumococcal conjugate vaccine 20 (PCV20) provides protection against 20 serotypes of *S. pneumoniae,* viz., 1, 3, 4, 5, 6A, 6B, 7F, 8, 9V, 10A, 11A, 12F, 14, 15B, 18C, 19A, 19F, 22F, 23F, and 33F.

Regimen: For those who have not previously received a dose of PCV13, PCV15, or PCV20 or whose previous vaccination history is unknown, one dose of PCV15 or one dose of PCV20 (Grade A, Level A). If PCV15 is used, administer one dose of PPSV23 at least 1 year after the PCV15 dose (may use a minimum interval of 8 weeks for adults with an immunocompromising condition) (Grade A, Level B).

Target Population: All adults aged 50 and older. Adults aged 19-64 with certain risk factors may need these vaccines earlier (Grade A, Level B).

Special Considerations: Pneumococcal vaccines protect against bacterial pneumonia and related infections, which can be serious in older adults. For adults with certain chronic health conditions like diabetes, heart disease, or chronic lung disease, LCVAC recommends PCV13 in series with PPSV23, usually administered a year after the PCV vaccination. For adults aged ≥18 years with an immunocompromising condition, CSF leak, or cochlear implant, the duration between the two vaccines has to be reduced to 8 weeks in special populations. PCV13 and PPSV23 should not be co-administered (Grade A, Level A) [35].

Shingles (Zoster) Vaccine

The shingles vaccine is effective in preventing herpes zoster and its complications, particularly postherpetic neuralgia (PHN) and herpes zoster ophthalmicus (HZO) [36]. The recombinant zoster vaccine Shingrix (GSK, London, UK) is recommended for adults aged 50 years and older, as well as those with weakened immune systems, providing over 90% protection against shingles and PHN [37]. The lack of specific mortality data highlights the need for improved surveillance and reporting mechanisms to better understand the impact of herpes infections on public health in India [38,39]. The live zoster vaccine has been shown to be effective in reducing the risk of herpes zoster (HZ), PHN, herpes zoster ophthalmicus (HZO), and hospitalization, although its protection wanes over time. Recent studies suggest the shingles vaccine may also lower the risk of dementia [40,41]. Overall, the shingles vaccine is a safe and effective way to prevent this painful and potentially serious disease. The vaccination landscape is dynamic, and MoHFW may consider including the shingles vaccine in future immunization programs based on epidemiological data, cost-effectiveness, and vaccine availability.

The recommendations of LCVAC for recombinant adjuvanted zoster vaccine are as follows.

Dosage, Administration, and Target Population: Administer two doses of recombinant zoster vaccine, 2-6 months apart, to all adults aged 50 years and older, regardless of prior history of herpes zoster or varicella vaccination. Shared decision-making is recommended for adults aged 19-49 years with immunocompromising conditions or other high-risk factors such as diabetes, chronic kidney disease (CKD), chronic obstructive pulmonary disease (COPD), HIV infection, hematologic malignancies, those with primary or secondary immunodeficiencies, and those needing chronic immunosuppressive therapy (Grade A, Level A).

Special Considerations: Shingles can be more severe in older individuals, and vaccination is recommended to reduce the risk of developing the disease (Grade A, Level B).

Hepatitis A and B Vaccine

The hepatitis B vaccine is effective in preventing hepatitis B virus (HBV) infection, which can lead to chronic liver disease and liver cancer. Recommended for all infants at birth and children up to 18 years, it is also advised for adults at high risk, including healthcare workers and those with certain medical conditions [42]. The prevalence of hepatitis B in India over the last five years has remained significant, with estimates indicating that approximately 2% to 4% of the general population is infected with the virus [43]. This translates to about 40 million carriers in the country, contributing to India harboring 10% to 15% of the global hepatitis B pool [44]. In tribal areas, prevalence rates can be much higher, reaching up to 15.9%. Despite efforts to combat this public health issue, including vaccination programs, vertical transmission from mother to child remains a major concern, accounting for a substantial number of new infections annually. The vaccine is administered in a three-dose series, achieving over 90% long-term immunity in recipients. Safety profiles indicate minimal side effects, primarily soreness at the injection site. The vaccine is considered one of the safest, with extensive global use since its introduction, significantly reducing HBV-related morbidity and mortality. Hepatitis B vaccine is included in the Universal Immunisation Programme (UIP), providing free vaccination to infants at birth and at multiple follow-up doses. The ministry emphasizes the importance of vaccinating pregnant women and administering hepatitis B immunoglobulin to newborns born to infected mothers.

The hepatitis A vaccine is a highly effective immunization that protects against hepatitis A virus (HAV) infection, primarily transmitted via the fecal-oral route. Recommended for children starting at 12 months, the vaccine is crucial for at-risk populations, including travelers to endemic regions, men who have sex with men, and individuals with chronic liver disease. The vaccine is available in both inactivated and live attenuated forms, demonstrating strong immunogenicity and safety. Studies indicate that immunity can persist for over 20 years post-vaccination. Hepatitis A vaccination has significantly reduced disease incidence globally, highlighting its importance in public health strategies to prevent outbreaks. The MoHFW may consider introducing hepatitis A vaccination for specific high-risk groups in the future, such as international travelers or healthcare workers, based on epidemiological data and cost-effectiveness.

The recommendations of LCVAC are as follows.

Dosage and Administration Depending on Individual Risk Factors: For effective immunization against hepatitis A and B, single-antigen vaccine formulations should be administered in a two-dose schedule at 0 and 6-12 months (Grade B, Level B) [45]. If using the combined hepatitis A and hepatitis B vaccine, a three-dose schedule at 0, 1, and 6 months is recommended, or alternatively, a four-dose schedule can be followed with doses given on days 0, 7, and 21 to 30, followed by a booster at month 12 (Grade A, Level A) [46,47]. Hepatitis A immunization is particularly recommended for adults in high-risk groups, including those with chronic liver disease, men who have sex with men, individuals who use illegal drugs, those infected with other hepatitis viruses, recipients of clotting factor concentrates, individuals awaiting or who have received a liver transplant, and food handlers [48]. The live HBV vaccine should be administered subcutaneously at a dose of 0.5 mL. For hepatitis B vaccination in adults who have not been previously vaccinated, a three-dose series is advised: the second dose should be given one month after the first dose, and the third dose at least six months after the first [49]. The recommended dose for individuals over 18 years of age is 1 mL (20 µg), administered intramuscularly.

Target Population (Hepatitis B Vaccine): Adults at risk of hepatitis B exposure, including those with specific medical conditions or lifestyle factors (Grade A, Level A).

Target Population (Hepatitis A Vaccine): May be recommended based on travel plans or potential occupational exposure (Grade B, UPP).

Special Considerations (Hepatitis B Vaccine): Healthcare providers may assess the need for hepatitis B vaccination based on individual risk factors (Grade B, UPP).

Special Considerations (Hepatitis A Vaccine): Men who have sex with men (MSM), people with chronic liver disease, people with clotting factor disorders, and individuals with compromised immune systems (Grade A, Level B).

Meningococcal Vaccine

The meningococcal vaccine is a critical tool for preventing invasive meningococcal disease, a serious bacterial infection that can lead to meningitis and septicaemia [50]. The prevalence of invasive meningococcal disease in India over the last five years has been characterized by significant underreporting and unreliable data due to inadequate surveillance systems. Meningococcal disease is the third most common cause of bacterial meningitis in children under five, accounting for approximately 1.9% of all cases. A systematic review indicated a prevalence of 12.1% during epidemics and 0.76% in endemic conditions, with a case fatality ratio (CFR) of 12.8% in epidemic settings. Serogroup A is primarily responsible for reported cases, although non-A serogroups have also been documented [51,52]. Despite an estimated 3,000 endemic cases annually, the actual burden remains poorly defined, necessitating improved surveillance and vaccination strategies to address the disease effectively. There are several types of meningococcal vaccines available, including those that protect against groups A, B, C, W, and Y [53]. In the UK, all babies are offered the MenB (meningococcal vaccine against serogroup B) and MenC (meningococcal vaccine against serogroup C) vaccines as part of their routine immunizations, with a booster dose of MenACWY given at around 14 years of age [54]. The MenACWY vaccine is also recommended for travelers to certain countries, such as Saudi Arabia and sub-Saharan Africa. Meningococcal vaccines have significantly reduced the incidence of meningococcal disease in countries where they are widely used. However, ongoing surveillance and targeted vaccination strategies are crucial to further reduce the global burden of this devastating disease. The MoHFW may monitor meningococcal disease cases to assess the disease burden and potential need for vaccination programs in the future.

The recommendations of LCVAC are as follows.

Dosage and Administration: Meningococcal conjugate vaccines are recommended to protect against invasive meningococcal disease, particularly in high-risk populations. CDC recommends that all 11- to 12-year-old adolescents receive a MenACWY vaccine. Since protection wanes, CDC recommends a MenACWY booster dose at age 16 years. The booster dose provides protection during the ages when adolescents are at highest risk The Indian Academy of Pediatrics (IAP) advises administering the quadrivalent meningococcal conjugate vaccines (MenACWY - meningococcal vaccine against serogroups A, C, W, and Y) to children and adolescents, with a primary series consisting of one dose for those over two years of age and two doses for children aged 9-23 months, spaced at least three months apart (Grade A, Level B) [55,56]. A booster dose is recommended every five years for individuals at continued risk of exposure. High-risk groups, including those with complement deficiencies, asplenia, or HIV, should receive a two-dose primary series with an eight-week interval, followed by boosters every five years (Grade A, Level B). Vaccination is also recommended during outbreaks and for travelers to endemic regions. For healthy adults traveling to endemic regions (e.g., sub-Saharan Africa, Saudi Arabia, etc.), a single dose of MenACWY is recommended 10-14 days before travel. Booster every 5 years if continued risk.

Target Population: Meningococcal vaccines are recommended for children aged 2 years and older with certain medical conditions, such as terminal complement component deficiencies, functional or anatomical asplenia, and those with HIV [57, 58]. A two-dose primary series of meningococcal conjugate vaccine (MCV) is recommended for high-risk individuals, administered 8 to 12 weeks apart.

Special Considerations: The risk of meningococcal disease tends to increase with age, and individuals with certain medical conditions or a weakened immune system may be at higher risk. During outbreaks, particularly of serogroup A meningococci, the use of MCVs is advised.

Haemophilus influenzae type b (Hib) Vaccine

The *Haemophilus influenzae *type b (Hib) vaccine is a highly effective immunization that prevents severe infections caused by Hib, including meningitis and pneumonia. Introduced in the late 1980s, the conjugate vaccine has led to a dramatic decline in Hib disease incidence, reducing cases by over 90% in vaccinated populations [59, 60]. Recommended for infants starting at six weeks of age, the vaccination schedule typically includes three doses, with a booster at 12-15 months. The vaccine is safe, with mild side effects such as injection site pain and fever. Its widespread use has significantly decreased Hib-related morbidity and mortality, making it a cornerstone of pediatric vaccination programs globally. By including the Hib vaccine in the UIP, India has demonstrated its commitment to providing comprehensive immunization coverage for infants and protecting them from life-threatening diseases.

The recommendations of LCVAC are as follows.

Dosage and Administration: Administer a single dose of Hib conjugate vaccine to adults with functional or anatomic asplenia, sickle cell disease, hematopoietic stem cell transplant recipients, HIV infection, certain hematologic malignancies, and long-term immunosuppressive therapy who were not vaccinated in childhood (Grade A, Level B). Routine Hib vaccination is not indicated for healthy adults (Grade A, Level A). The Hib conjugate vaccine is crucial for preventing invasive Hib disease, particularly in young children. The recommended immunization schedule includes a primary series starting at 2 months of age, with doses administered at 2 months, 4 months, and 6 months if using vaccines like ActHIB (Sanofi Pasteur, Paris, France); Hiberix (GlaxoSmithKline Biologicals, Rixensart, Belgium); Pentacel (Sanofi Pasteur, Paris, France); Vaxelis (Sanofi Vaccines, Toronto, Canada). If PedvaxHIB (Merck Sharp & Dohme LLC, New Jersey, USA) is used, a two-dose series is given at 2 months and 4 months [61]. A booster dose is recommended between 12 and 15 months of age, administered at least 8 weeks after the last dose of the primary series. For children aged 15 months or older who are receiving the vaccine for the first time, only one dose is necessary. Additionally, certain high-risk groups may require additional doses depending on their health status.

Target Population: Generally not routinely recommended for healthy adults. However, those with specific medical conditions or functional or anatomic asplenia may be advised to receive the vaccine.

Special Considerations: Hib disease is more commonly a concern for young children, and routine vaccination during infancy has significantly reduced the incidence in many regions (Grade A, Level A).

Recommendations for human papillomavirus (HPV), measles, mumps, and rubella (MMR), varicella, and travel vaccines (Japanese encephalitis, rabies, yellow fever) follow standard CDC/WHO/Indian guidelines and are summarized in Table 1. A detailed discussion is beyond the scope of this article.

**Table 1:** LCVAC vaccine recommendations, target population and special considerations LCVAC: Life Course Vaccination Continuum; Tdap: Tetanus, Diphtheria, and Pertussis; Td: Tetanus and Diphtheria toxoid (booster); HPV: Human Papillomavirus; PCV13, PCV15, PCV20 — Pneumococcal Conjugate Vaccine (13-, 15-, 20-valent, respectively); PPSV23: Pneumococcal Polysaccharide Vaccine (23-valent); MenACWY: Meningococcal vaccine (serogroups A, C, W, Y); MenB: Meningococcal serogroup B vaccinbe; MenA: Meningococcal polysachharide serogroup A vaccine; MSM: Men who have sex with men; Hib: Haemophilus influenzae type b; MMR: Measles, Mumps, and Rubella; HBV: Hepatitis B Virus

Target Population	Recommended Vaccine	
	Core Vaccines	Additional Vaccines
Adults aged 18 to 60 years with co-morbidities, including immunocompromized adults	Tetanus, Diphtheria, and Pertussis (Tdap): Recommended once every 10 years as a booster dose. Influenza: Annual vaccination strongly recommended, prioritising those at high risk for adverse outcomes. Hepatitis B: Recommended for everyone who is not already immune. Pneumococcal vaccine: For adults >50 years and 18-49 years with certain chronic health conditions like diabetes, heart disease, or chronic lung disease.	Meningitis: Depending on travel plans and specific conditions, meningococcal vaccines (MenACWY, MenB, or polysaccharide MenA) may be needed. Hepatitis A: Depending on travel plans or potential occupational exposure. Zoster (Shingles): Recommended for those >50 years. May be recommended based on individual risk factors and specific health conditions. HPV: Catch-up vaccination may be recommended depending on age, vaccination history, and specific health conditions.
Elderly above 60 years of age	Tetanus, Diphtheria, and Pertussis (Tdap): One dose of Tdap is recommended for adults who have not previously received it, followed by Td boosters every 10 years, especially for those in close contact with infants. Influenza: Annual vaccination is strongly recommended for all adults aged 65 and older. Pneumococcal: PCV15 or PCV20 for all adults 50 years or older. Older adults vaccinated with PCV13 at any age and PPSV23 at or after the age of 65 have an option to get PCV20 or not get any additional pneumococcal vaccines.	Shingles (Zoster): Recombinant Adjuvanted Zoster Vaccine for adults aged 50 and older. Hepatitis B: May be recommended depending on individual risk factors and healthcare provider advice. Meningococcal Vaccination: Adults aged 55 and older, especially those with certain medical conditions or risk factors, are advised to receive meningococcal conjugate vaccines in some countries. Hepatitis A: May be recommended for specific groups, such as MSM, those with chronic liver disease, clotting factor disorders, or compromised immune systems, based on travel plans or occupational exposure. Haemophilus influenzae type b (Hib) Vaccination: Generally, not routinely recommended for healthy adults, but may be advised for those with specific medical conditions or functional or anatomic asplenia.
Healthy adults aged 18 to 60 years, including pregnant women and travellers	Tetanus, Diphtheria, and Pertussis (Tdap): Recommended once every 10 years as a booster dose. Influenza: Annual vaccination recommended for everyone, especially pregnant women and healthcare workers. Special Population Pregnant Women: Influenza (Flu) Vaccine: It is recommended during flu season for pregnant women to protect both the mother and the baby. Tdap (Tetanus, Diphtheria, Pertussis) Vaccine: Recommended during each pregnancy, ideally between 27 and 36 weeks of gestation. Hepatitis B Vaccine: If not previously vaccinated, it may be recommended during pregnancy. Travel: Consider influenza, rabies, MenB, Japanese encephalitis, and yellow fever vaccines.	Human Papillomavirus (HPV): Recommended for men and women through age 26 (catch-up for older adults under certain circumstances). Meningitis: Depending on travel plans, meningococcal vaccines (MenACWY, MenB, or polysaccharide MenA) may be needed. Hepatitis B: Recommended for everyone who is not already immune. Hepatitis A: May be recommended depending on travel plans or potential occupational exposure. Zoster (Shingles): Recommended for adults 50 and older. Adults aged 50–59 years without comorbidities should receive the zoster vaccine as part of routine preventive care, not only those with comorbidities. Pneumococcal: Recommended for adults 65 years and older, and those with certain chronic health conditions. Measles, Mumps, Rubella (MMR) Vaccine: Typically given in two doses, especially if not previously vaccinated. Varicella (Chickenpox) Vaccine: If the individual has not had chickenpox or received the vaccine in childhood, it may be recommended.

LCVAC consensus: recommendations for vaccine awareness, education, and vaccination centres in India

The consensus on adult vaccination in India by the LCVAC members can be summarized as follows.

Acknowledgment of Challenges: Members recognized access issues, lack of confidence in vaccines, hesitancy, cost, and inadequate prioritization by healthcare providers as barriers to adult vaccination [62, 63].

Systemic Changes Needed: There was agreement on the necessity for policymakers' involvement, improving affordability and accessibility to vaccines, and changing prescription practices among healthcare providers to promote adult vaccination. Strengthening surveillance systems and improving adult immunization data collection are also essential for identifying disease burden, monitoring vaccine uptake, detecting gaps in coverage, and guiding evidence-based vaccination policy. The development of a national adult immunization registry and integration of adult vaccination data into existing health information systems may support more accurate tracking, reporting, and planning of life-course vaccination programs in India.

Advocacy and Culture Shift to Value-Based Care: LCVAC recommends that the MoHFW: (a) create a national adult immunization registry; (b) mandate Tdap boosters for healthcare workers; (c) require influenza vaccination for pregnant women as part of routine antenatal care. Efforts to advocate for vaccination, promote a culture of vaccination, and lead by example were deemed essential to increase vaccination uptake. Involvement of influencers was considered useful for the dissemination of the message.

Education and Awareness: Emphasizing the importance of vaccination, addressing misconceptions, and standardizing messaging across healthcare providers. Health Care Workers' (HCW) sensitization for erasing the disconnect between their perceptions and practices needs to be undertaken through consistent and regular advocacy. 

Collaborative Approach: Collaboration among stakeholders, including government, healthcare providers, private organizations, and professional societies, was seen as crucial for effective vaccination programs.

Following the needs assessment, the LCVAC will embark on evidence-based deliberation. By leveraging global best practices, scientific literature, and expert consultations, the committee shall evaluate potential interventions, including the introduction of new vaccines, optimizing schedules, and addressing access barriers. Stakeholder consultations with healthcare professionals, community leaders, and civil society organizations shall provide invaluable insights and ensure policy inclusivity.

The draft will undergo rigorous public scrutiny, with feedback meticulously analysed and incorporated to ensure transparency and public trust before final submission to government authorities for approval. The LCVAC policy document would include guidelines on vaccine recommendation in adults, identification of the target population, vaccination schedule, access, education, and outreach [12,62-65]. This collaborative approach shall pave the way for effective implementation and, ultimately, a healthier and more resilient India. Overall, Life Course Vaccination Continuum Advisory Board members pledged to foster the life-course approach to immunization as described in the following sections.

Vaccine Awareness

Recognizing the role of immunization as a strategy to prevent diseases and maximize health over one’s entire life, with a specialized focus on adult and geriatric immunization. Improving public and provider awareness within 2 years (by 2027) is a critical component of achieving this goal. Vaccine hesitancy, driven by misinformation and a lack of confidence, hinders the achievement of optimal vaccination rates [66, 67]. It is crucial to address these concerns by employing evidence-based communication, engaging with the community (by tailoring messages for different literacy levels or languages in India), and resolving financial obstacles.

In this context, leveraging modern communication tools becomes essential. The use of social media platforms, mass media campaigns, and digital health technologies can significantly enhance the reach and impact of accurate vaccine-related information. These platforms enable the timely dissemination of scientifically validated messages, counter misinformation, and facilitate interactive engagement with both the public and healthcare providers.

Life Course Vaccination Continuum recommends a multi-lingual, multi-channel awareness campaign targeting adults aged 50 years and older through: (a) television and radio public service announcements in major Indian languages; (b) Accredited Social Health Activist (ASHA) and community healthcare worker toolkits for door-to-door education and counselling; and (c) social media campaigns targeting younger adults to promote intergenerational awareness and parental vaccination uptake. These campaigns should be coordinated jointly by the Ministry of Health and Family Welfare, state health departments, and professional medical societies.

Life Course Vaccination Continuum recommends that the Indian Council of Medical Research (ICMR) support multicentre studies on adult vaccine effectiveness, immunosenescence, vaccine uptake patterns, and real-world implementation outcomes in Indian populations by 2028, to strengthen evidence-based adult immunization policies in India. Adults require protection against diseases such as influenza, shingles, and tetanus, and periodic reinforcement of childhood vaccinations may be necessary to maintain lasting immunity [68,69].

The panel recommends strategies for enhancing vaccination coverage across the following areas: dedicated resource allocation for adult vaccination initiatives; addressing vaccine hesitancy and building confidence; ensuring access to vaccines; optimising the organization and management of vaccination programmes; strengthening immunization information systems; and aligning with health policy [70,71]. LCVAC recommends that state governments progressively allocate dedicated funding for adult vaccination procurement, outreach activities, healthcare worker training, and immunization infrastructure strengthening within their public health budgets by 2028. Priority investments should include vaccine supply chains, cold-chain expansion, adult vaccination centres, and digital immunization registries to improve vaccine accessibility and coverage across India.

Enhancing the extent of vaccination is essential for safeguarding individuals and communities from avoidable illnesses [72]. Addressing vaccine hesitancy requires understanding the reasons behind it, employing effective communication strategies, combating misinformation, increasing vaccine accessibility, and fostering community engagement [70,73,74]. Reducing cost barriers, expanding service availability, implementing reminder systems, and leveraging technology can help increase access and convenience. Building local partnerships, empowering community champions, and investing in education are crucial for community engagement and empowerment [75]. By implementing these strategies, healthcare professionals and public health initiatives can promote vaccine acceptance and protect public health.

Vaccine Education

The key elements of a successful educational campaign for healthcare professionals in advanced vaccinology and epidemiology include conducting a needs assessment to identify the educational requirements of the target audience through surveys, focus groups, or interviews. Advanced vaccinology and epidemiology training is essential for effective vaccine education, equipping individuals with the knowledge to understand immunological principles, evaluate vaccine safety and efficacy, develop vaccination programmes, and communicate effectively about vaccines. Training healthcare providers on adult vaccination over 3-5 years (by ~2030), measured through indicators such as the number of trained providers, should be an integral objective of such campaigns. The campaign should have clear, measurable, and achievable objectives, with content based on the latest scientific evidence and best practices. Additionally, the delivery format should be engaging and interactive to capture the audience's interest. Finally, evaluating the campaign is essential to assess its effectiveness in achieving its objective [76].

Vaccination Centres

Ensuring equal access to vaccination centres across India, regardless of geographical or socioeconomic differences, is both a crucial public health necessity and a moral duty. Establishing vaccination centres within 5 years, measured through indicators such as the number of centres established, is a key goal in achieving this vision. The panel acknowledges that a comprehensive strategy is needed to address obstacles related to geography, socio-economic disparities, and cultural considerations. This includes extending immunization centres into rural areas and utilising mobile units to reach isolated communities [77,78]. These centres must be equipped with appropriate cold chain storage (including -20°C freezers for recombinant zoster vaccine where applicable) and trained staff for vaccine handling.

Financial barriers must be tackled through government initiatives, involvement of non-governmental organisations, and public-private partnerships. Furthermore, addressing vaccine hesitancy requires transparent communication and community engagement. By integrating geographical expansion, financial inclusivity, cultural sensitivity, and technological empowerment [79], we can create a framework that guarantees access to vaccinations for all individuals in India. Additionally, investing in staff training on vaccination protocols and community outreach initiatives is crucial for enhancing public awareness and trust in immunization efforts, ultimately safeguarding public health [80].

The consensus discussion focused on the implementation of adult vaccination guidelines within the private sector. While government vaccination programs in India have predominantly emphasized paediatric immunization, vaccination across the entire lifespan is essential. To address this gap, a life-course approach to vaccination has been introduced, promoting immunization at all stages of life.

Limitations

This consensus has several limitations. First, while a systematic literature search was conducted, the quality and quantity of Indian-specific data on adult vaccine-preventable diseases remain limited, particularly for herpes zoster, meningococcal disease, and pertussis in adults. Second, the expert panel, though multidisciplinary, did not include patient representatives or health economists, which may have influenced the prioritization of recommendations. Third, the modified Grading of Recommendations, Assessment, Development and Evaluation (GRADE) system used here relies on expert interpretation of evidence; formal GRADE profiler (GRADEpro) or GRADE Confidence in the Evidence from Reviews of Qualitative (GRADE-CERQual) assessments were not performed. Fourth, cost-effectiveness analyses were not systematically reviewed, and recommendations may not be feasible in all resource settings across India. Fifth, the consensus did not address adolescent vaccination (ages 10-17) in detail, nor did it cover emerging vaccines such as respiratory syncytial virus (RSV) or newer pneumococcal conjugate formulations (PCV15, PCV20) in depth. Finally, implementation timelines and targets are aspirational and require validation through pilot studies.

## Conclusions

The LCVAC consensus focuses on adult immunization in India through routine vaccination recommendations with Tdap booster (including every pregnancy), annual trivalent influenza vaccination, age and risk-based pneumococcal vaccination, two-dose recombinant zoster vaccine for adults ≥ 50 years, risk-based hepatitis A and B vaccination, meningococcal vaccination for high-risk individuals, including travellers, and Hib vaccination for high-risk adults. Furthermore, this consensus underscores that to improve adult vaccination in India, there is a need to address barriers such as access, hesitancy, affordability, and inadequate prioritization, while aligning with the life-course approach. Implementation will proceed through three parallel pillars: public awareness campaigns, healthcare professional education in advanced vaccinology, and the establishment of dedicated adult vaccination centres nationwide.

The Indian government has actively engaged in vaccination policy formulation and implementation, collaborating with the public and private sectors to improve vaccine accessibility and affordability. Community engagement at the grassroots level is crucial for building consensus and promoting vaccination uptake. Future plans involve strengthening vaccine manufacturing capabilities, enhancing vaccination infrastructure, developing a robust supply chain, and leveraging technology to streamline the vaccination process. Tailored strategies and outreach programs will ensure consideration of vulnerable populations' needs in vaccination policies. To reduce financial barriers to adult vaccination, LCVAC recommends the inclusion of priority adult vaccines within publicly funded insurance schemes such as Ayushman Bharat-Pradhan Mantri Jan Arogya Yojana (PM-JAY) and relevant state health insurance programs. The panel also supports tax incentives for employers implementing workplace vaccination programs, subsidized vaccination drives for economically vulnerable populations, and differential or tiered vaccine pricing strategies based on income categories to improve equitable vaccine access.

Re-evaluating immunization programmes through the lens of an LCVAC presents a strategic opportunity to optimize vaccination services, thereby enhancing health outcomes for both individuals and communities in India. By recognizing that vaccination is a lifelong necessity rather than merely a childhood priority, the LCVAC framework facilitates the integration of immunisation into broader health services. This holistic perspective enables the identification of gaps in coverage and the tailoring of interventions to meet the diverse needs of populations at various life stages. Ultimately, adopting an LCVAC can lead to improved vaccine uptake, greater health equity by systematically reducing disparities in access in rural areas, and a more resilient public health infrastructure.

## Appendices

Life Course Vaccination Continuum Advisory Board

1. Dr. Parvaiz Koul, Professor of Pulmonary Medicine and Director, Sher-I-Kashmir Institute of Medical Sciences, Srinagar

2. Dr. Raja Dhar, Director & HOD, Department of Pulmonology, CMRI, Kolkata.

3. Dr. Vivekanand Jha, Executive Director, The George Institute for Global Health, India

4. Dr. Vijay Kher, Chairman, Division of Nephrology & Transplant Medicine, Epitome Kidney & Urology Institute, New Delhi.

5. Dr. Vivek Nangia, Principal Director and Head, Institute of Respiratory, Critical Care & Sleep Medicine, Max Hospital, Delhi.

6. Dr Rahul Bhargava, Principal Director - Haematology & BMT, Fortis Memorial Research Institute, Gurugram

7. Dr. Mangesh Tiwaskar, Consultant Physician and Diabetologist, Shilpa Medical Research Centre, Mumbai.

8. Dr. Agam Vora, Senior Consultant, Pulmonology at Vora Clinic, Mumbai.

9. Dr. V. Ramasubramanian, HOD - Infectious Diseases, HIV & Tropical Medicine at Apollo Hospitals, Chennai.
